# Impact of a Cold Environment on the Performance of Professional Cyclists: A Pilot Study

**DOI:** 10.3390/life11121326

**Published:** 2021-12-01

**Authors:** Florence Riera, Samuel Bellenoue, Simon Fischer, Henri Méric

**Affiliations:** 1 Laboratory IMAGE (UMR ESPACE DEV, 228), University of Perpignan Via Domitia, 66000 Perpignan, France; s.fischer25@laposte.net (S.F.); henri.meric@univ-perp.fr (H.M.); 2 Wanty Gobert Cycling Team, 7500 Tournai, Belgium; sambellenoue@gmail.com

**Keywords:** cycling, athletes, weather parameters, core temperature, exercise performance, exercise thermoregulation

## Abstract

The practice of physical activity in a variable climate during the same competition is becoming more and more common due to climate change and increasingly frequent climate disturbances. The main aim of this pilot study was to understand the impact of cold ambient temperature on performance factors during a professional cycling race. Six professional athletes (age = 27 ± 2.7 years; height = 180.86 ± 5.81 cm; weight = 74.09 ± 9.11 kg; % fat mass = 8.01 ± 2.47%; maximum aerobic power (MAP) = 473 ± 26.28 W, undertook ~20 h training each week at the time of the study) participated in the Tour de la Provence under cold environmental conditions (the ambient temperature was 15.6 ± 1.4 °C with a relative humidity of 41 ± 8.5% and the normalized ambient temperature (T_awc_) was 7.77 ± 2.04 °C). Body core temperature (T_co_) was measured with an ingestible capsule. Heart rate (HR), power, speed, cadence and the elevation gradient were read from the cyclists’ onboard performance monitors. The interaction (multivariate analysis of variance) of the T_awc_ and the elevation gradient has a significant impact (F(1.5) = 32.2; *p* < 0.001) on the variables (cadence, power, velocity, core temperature, heart rate) and on each individual. Thus, this pilot study shows that in cold environmental conditions, the athlete’s performance was limited by weather parameters (ambient temperature associated with air velocity) and race characteristics. The interaction of T_awc_ and elevation gradient significantly influences thermal (T_co_), physiological (HR) and performance (power, speed and cadence) factors. Therefore, it is advisable to develop warm-up, hydration and clothing strategies for competitive cycling under cold ambient conditions and to acclimatize to the cold by training in the same conditions to those that may be encountered in competition.

## 1. Introduction

Professional road cycling races are composed of events ranging from one day to three weeks of competition with race durations ranging from less than one hour to several hours with varying race profiles [1]. Professional road races are races where the type of effort provided is intermittent [1]. The average power output is about 210 W for flat stages and 270 W for mountain stages, but power output alone is not sufficient as a performance factor [2,3,4]. 

For many authors [2,3,4] road cycling is mainly an outdoor endurance activity with multiple performance factors. Among the factors considered, some authors [5,6,7,8,9] have hypothesized that environmental factors such as temperature, solar radiation, environment, air velocity with the Wind Chill Index (WCI) or humidity can influence the level standard of performance in endurance sports such as cycling, triathlon, or running [8]. Indeed, these endurance sports sometimes take place in snow, rain, at negative temperatures or at average temperatures above 35 °C. Recent studies underline this [6,10,11,12].

It is commonly accepted today that performance in endurance and intermittent sports is impaired in an environment where endogenous heat production exceeds the body’s thermoregulatory capacity [13,14] because the alteration of thermal homeostasis leads to the development of compensatory mechanisms that impair performance. 

Thermoregulation mechanisms are the set of processes that allow humans to maintain their internal temperature within normal limits regardless of their metabolic level or the temperature of the surrounding environment. It is based on a constant balance between heat input and heat loss. The skin exchanges heat with the surrounding environment and the direction and intensity of these exchanges depends on the temperature of the environment and the thermal insulation capacity of the skin. There are four modes of heat exchange between the skin and the surrounding environment: radiation, convection, conduction, and evaporation. In a hot environment, the combination of heat and exercise leads to an increase in physiological stress greater than that induced by either stress individually [15]. The dissipation of body heat during exercise in an ecological environment is influenced by the convection of the air, the humidity of the environment and the speed of movement of the athlete. But this dissipation in the environment, is not linear [16]. At high speeds, convection is high and the temperature felt by the athletes is lower than the real temperature. On the other hand, convection has less influence when the speed decreases, which is the case in cycling in the ascending sections (climb) of races [17]. Conversely, in a cold environment, this heat dissipation can become harmful since in these conditions the main problem in terms of thermoregulation is to limit heat loss. Convection also depends on the exposed body surface. Thus, the front of an exercising cyclist (head, arms, trunk and legs) is more exposed to wind speeds close to its speed of travel [17]. While at the back of the body the air movements are weaker.

The relationship between a cold environment (temperature less than or equal to 18 °C in the water or >8 °C in the air) and performance is less documented than the relationship between performance, although authors have focused on thermal physiology and the impact of a cold environment on performance in diving [18,19] in cross-country skiing [20,21] or in marathon [8]. These show there is a U-shaped relationship between athletic performance and heat stress, indicating that temperatures between 8 and 14 °C are the best for maximizing endurance performance in marathon running. On the other hand, any temperature about or below this range reduces running performance. For example, Sandsund et al. [22] observed laboratory based cross country skiing performance was better at −4 °C and 1 °C than at −14 °C, 10 °C and 20 °C. Here, rectal temperature increased but skin temperature decreased with cold exposure and the vasoconstriction mechanisms that take place to avoid heat loss between the body and the ambient air. This confirms the work of Galloway et al. [14] who describe the relationship between temperature environmental and performance capacity in an inverted U shape. According to these authors, endurance performance is negatively affected in both cold and warm conditions with the existence of an optimal temperature for endurance performance appears to be optimal when core temperature (T_co_) (reflected by rectal temperature (T_re_) or gastrointestinal temperature (T_gi_)) is 39.2 °C, whereas it is impaired for a T_co_ of 38.8 °C. This agrees with the study by Parkin et al. [23] which shows that in the laboratory, submaximal performance (time to exhaustion at 70% VO_2max_) is better in cold (3 °C) conditions compared to the temperate (20 °C) or warm (40 °C) condition. The Tre reached at the end of the test is 39.2 °C for the cold and neutral conditions while it is higher than 39.5 °C for the warm condition. 

In addition to physiological demands (i.e., high aerobic capacity and power with emphasis on anaerobic-to-aerobic transition) [24,25] and tactical considerations (i.e., influence of position in the mass start) [26], road cycling races are conducted over different geographical profiles (e.g., uphill, flat, downhill), which are themselves subject to variation due to uncontrollable environmental factors (e.g., % slope elevation, temperature and/or relative humidity, wind speed). Also, pedaling in a cold environment has both a positive and negative impact on exercise performance. Compared to a temperate environment (18–25 °C), cycling in a cold environment (15 °C) poses serious challenges to human regulatory systems [14,27,28] which hinders endurance exercise performance [e.g., shorter time to exhaustion, longer time to completion of the event] [14,29,30] in response to the development of increased thermogenesis to compensate for environmentally related heat loss. Furthermore, it appears that performance impairment is dependent on the duration of the event, with longer ones involving greater degradation [31]. In real condition, this is particularly true where athletes are unable to compensate for the combination of endogenous (exercise-induced) and environmental heat stress [32,33,34,35,36].

The aim of this study is to investigate the impact of a cold environment on the thermoregulatory system, physiological factors and on the performance of professional road cycling in real exercise conditions. We hypothesized that a cold environment for a cycling race result in decreased performance due to the difficulty in achieving an optimal core temperature for endurance exercise. 

## 2. Materials and Methods

### 2.1. Participants

Six volunteer male professional cyclists from the Wanty Gobert Pro Cycling Team (Belgium), who participate in all the competitions of the season, were recruited to participate in this study (age = 27 ± 2.7 years; height = 180.86 ± 5.81 cm; weight = 74.09 ± 9.11 kg; % fat mass = 8.01 ± 2.47%; maximum aerobic power (MAP) = 473 ± 26.28 W). Athletes undertook ~20 h training each week at the time of the study. All athletes completed a medical questionnaire, were informed by an information letter and signed a written consent in accordance with the Declaration of Helsinki. They were previously interviewed by the team physician to determine the athletes’ suitability to participate in the study and to ingest the thermal capsule (BodyCap e-Celsius^®^, Caen, France).

### 2.2. Study Design (Procedure)

The study took place from February 16 during the Tour de la Provence from February 13 to 17, 2019. This day was chosen because it was the longest stage of the tour and the day before the riders had only a time trial. On the study day, athletes ate a standardized breakfast (1400 kcal) with 250 mL of beverage (tea or coffee), 250 mL of fruit juice and meat (oat, cheese, bread, jam and butter, egg) and lunch consisting of food (150 g of rice and 150 g of fish) and 250 mL energy drinks (water, maltodextrin, sodium). The experimental trial began at the same time of day for each athlete (between 1:00 and 5:30 p.m.) to control for circadian variations in temperature control and digestion. During the experimental trial, athletes wore cycling shorts (Iclu(clo) = 0.08), a chest heart rate monitor, a short-sleeved undershirt (Iclu(clo) = 0.08) short-sleeved jersey (Iclu(clo) = 0.36) and cycling jersey (Iclu(clo) = 0.36), socks (Iclu(clo) = 0.01), shoes (Iclu(clo) = 0.02) and a helmet. According to ASHRAE (www.ashrae.org, accessed on 29 October 2021) we can estimate the clothing insulation of the typical clothing ensemble worn by the cyclists participating in the study at 0.91 clo without the helmet.

Environmental conditions during the race were cold and dry (ambient temperature [Ta]: 15.6 ± 1.4 °C with normalized ambient temperature (T_awc_) at 7.77 ± 2.04 °C, humidity [H]: 41.5 ± 8.5%, wind velocity 7.5 ± 2.5 Km/h, Pression (hPa) 1031 ± 1.6 hPa and UV index = 2).

During the study, athletes were subjected to the Wind Chill Index (convection and velocity of the ambient air) which has the effect of increasing the sensation of cold on the skin, (T_awc_ = 7.77 ± 2.04 °C). Heart rate (HR) was recorded continuously during the race (HRM^®^ belt, Garmin, Olathe, KS, USA). Core temperature (T_co_) was collected every 30 s by gastrointestinal temperature using ingestible capsules. Athletes ingested the capsules 4–5 h before the start of the study to ensure that the capsule was in the duodenum thus avoiding variability in T_co_ due to pill movement or food/liquid consumption. 

Speed (m·s^−1^), power (W), cadence (rpm), distance (Km) and elevation gain (m) were recorded by cyclists’ onboard performance monitors (gps, power sensors, and barometric altimeter) at a recording frequency of one point per second (Garmin Edge 520).

**Figure 1:**
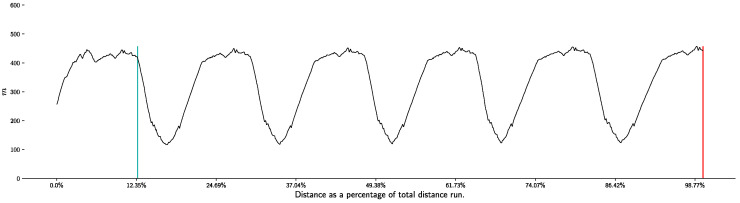
Profile of the race, section (red: downhill, light grey: uphill, light blue: flat) and lap (5 laps). A dummy start and a real start precede the stage of the race.

The characteristics of the event used in the study are described in Table 1. This stage is part of a stage competition. It took place during the third stage, which consisted of 5 laps of a circuit with uphill, downhill and flat, as shown in Figure 1. This means that it is preceded by a prologue (9.8 km two day before) and a road stage (191.6 km one day before). The day after the stage under study, the athletes participated in a final road stage (162.2 km) (Table 1).

The race was divided into three parts with respect to the terrain characteristics. It was thus designated as a downhill area, a flat area, and an uphill area (Figure 1).

### 2.3. Equipment and Measurements

Ambient temperature (°C) and wind (average speed [km·h^−1^] and gusts [km·h^−1^]) were recorded at the start, during, and at the finish of the competition (www.meteociel.fr (accessed on 16 February 2019)) and compared to data provided by the athletes’ sensors (Garmin Edge 520^®^).

The felt ambient temperature, expressed in °C, was normalized by the original windchill index (WCI) formula of Woodson modified by Parsons [37] using chilling temperature equation. The wind chill index (WCI) can be described as the cooling power of the atmosphere, combined with the effects of air temperature and air speed into a single index:

WCI = 1.16 (10 √v + 10. 45 − v) (33 − ta)

where ta is the air temperature in degrees Celsius and v is the wind and cyclist speed in m·s^−1^.

Being in a real condition we have come as close as possible to the indices taken into account by the sports team in their performance research. That is why we used this index. With an ambient temperature of 15.6 ± 1.37 °C corresponding to a T_awc_ of 7.77 ± 2.04 °C on average, the race took place in a cold environment. 

Although the athletes started the race wearing cuffs and a windbreaker jacket, all of them left these accessories during the race and ran most of the race wearing a short-sleeved undershirt, a short-sleeved jersey, and shorts as well.

Core temperature (T_co_) was recorded, before and during the run. While nacked body mass was assessed (±0.1 kg) before and after the run (SC 330P^®^, Tanita, Amsterdam, The Netherlands). Then an indication of hydration status throughout the study was determined by changes in nacked body mass. Heart rate was measured throughout the race using a chest sensor (Garmin HRM^®^ Belt) and the data was downloaded after the stage finish.

Athletes’ bikes were equipped with GPS sensors (Garmin Edge 520^®^; recording rate of one point per second) from which distance (Km), elevation gain (m), travel speed (m·s^−1^), power output (W) and cadence (rpm) were extracted.

Raw power data (power) were normalized by reporting as a percentage of a 20-min maximal test (functional threshold power test—FTP) [38,39] and reported to the athletes’ body weight (W·kg^−1^) to eliminate interindividual differences. 

The elevation gradient which is the derivative of the altitude was calculated to normalize the data between Athletes. This provides the elevation change per second. In our study, the elevation gradient reflects the increase in altitude. 

### 2.4. Statistical Analysis

Normality was tested using the Shapiro-Wilk’s test. The different variables were normalized to eliminate interindividual differences. The impact of normalized ambient temperature, elevation gradient, and the interaction between these two variables on: core temperature, heart rate, speed, power, cadence, was analyzed using multivariate analysis of co-variance (MANCOVA) with repeated measures. Effect size is reported by epsilon squared (ε^2^) [40], interpretation had been performed following the guidelines first establish by Cohen [41].

Pairwise comparisons were made for speed, power, cadence and core temperature to compare values across sections and laps, using Tukey-Kramer Test [42,43].

A correlation matrix was constructed for all variables over the entire stage in the first instance, and then for the different sections of the circuit, namely flat, uphill and downhill, in the second instance. 

Data analysis was performed with R studio software (PBC, Boston, MA, USA). All data are presented as mean (M) ± standard deviation (SD); where necessary, the significance level was set at 0.01. 

## 3. Results

Time, elevation gradient, heart rate, cadence and speed are presented for each section and per lap in Table 2. 

**Figure 2:**
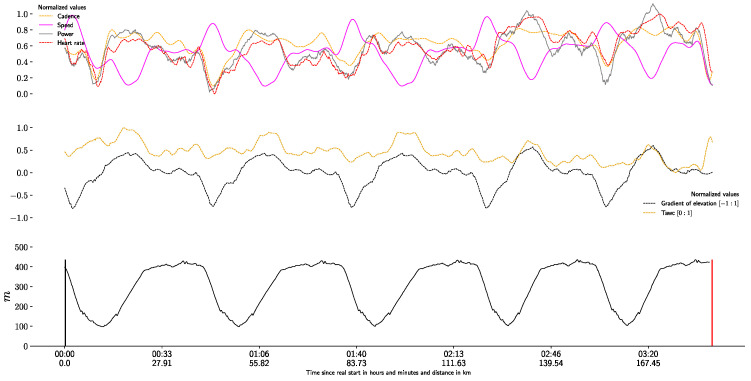
Individual example of variations in speed, cadence, ambient temperature normalized by WCI (T_awc_), heart rate (HR) and power during the test (normalized in a range of 0 to 1). The elevation of the route in meters is in black. The start and end of the run are indicated by a black and red vertical line respectively.

In Figure 2, we plot the speed, power, heart rate, and ambient temperature for an athlete in the event from the actual start to the finish line. 

### 3.1. Normalized Ambient Temperature (T_awc_) 

**Figure 3:**
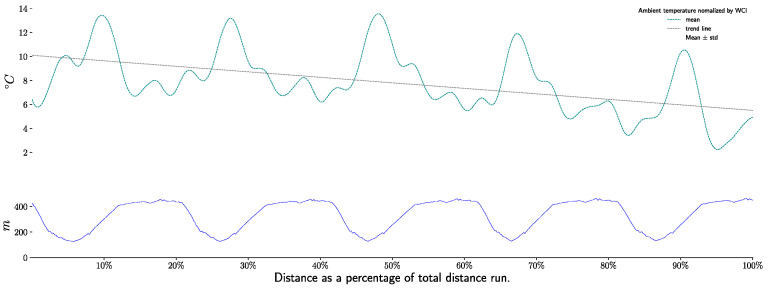
*Normalized ambient temperature (T_awc_)* during race, per sections and laps.

The ambient temperature during the test was 15.6 ± 1.37 °C. After being normalized with the windchill index, it turns out that the normalized outdoor temperature during the test was 7.77 ± 2.04 °C. As shown in Figure 3, T_awc_ decrease during the race. 

MANCOVA analysis shows that outdoor temperature influences all selected variables (normalized core temperature, normalized heart rate, normalized power, normalized cadence, and normalized speed) significantly (*p* < 0.001). Furthermore, this analysis reveals the influence of core temperature on the variables considered in isolation. 

Thus, outdoor temperature influenced core temperature (F(1) = 121.3; *p* < 0.001; ε^2^ = 0.06 small), heart rate (F(1) = 207.2; *p* < 0.001; ε^2^ = 0.09 medium), power (F(1) = 2634.9; *p* < 0.001; ε^2^ = 0.23 medium), cadence (F(1) = 2479.2; *p* < 0.001; ε^2^ = 0.04 small), and speed (F(1) = 26,999.0; *p* < 0.001; ε^2^ = 0.51 moderate).

Correlations between T_awc_ and power, power as ratio of ftp, speed, cadence, heart rate and core temperature were reported in Table 3. 

### 3.2. Core Temperature (T_co_)

**Figure 4:**
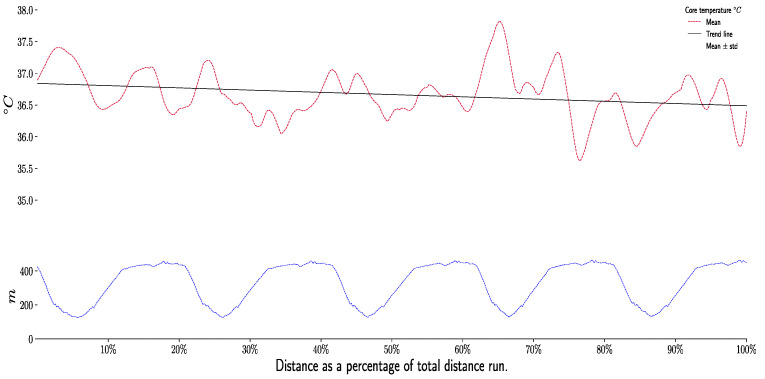
Evolution of the core temperature during the race per sections and laps. Values are represented by median and quartiles, using boxplot graph.

The core temperature decreases during the race, for each section and during each lap (Figure 4). This decrease is significant (for all pairwise, *p* < 0.001). Start core temperature was 37.32 ± 1.31 °C while final core temperature was 36.5 ± 1.44 °C. 

The correlation matrix shows that core temperature and heart rate are highly related in the uphill sections than flat or downhill one’s. No correlation link appears between core temperature and power output.

### 3.3. Heart Rate (HR)

According to the data in Table 2, heart rate increases during the run race in all sections and laps. Heart rate is significantly different between sections (downhill vs. uphill vs. flat, all pairs *p* > 0.001) and between laps *p* < 0.001) with the exception of laps 2 and 3 which show no difference between them.

### 3.4. The Body Mass 

Pre- and post-stage mass data revealed that all athletes lost weight during the event (−0.8 ± 0.6 kg). This corresponds to a loss of 1.2 ± 0.8% of the initial body weight. The largest loss was 2.04% of initial weight.

### 3.5. Elevation Gradient

The MANCOVA analysis shows that the elevation gradient has an influence on all the selected variables (normalized cadence, normalized power, normalized speed, normalized T_co_, normalized heart rate) in a significant way (*p* < 0.001). The elevation gradient also influences the selected variables taken in isolation, with for T_co_ (F(1) = 24.5; *p* < 0.001; ε^2^ = 0.01 very small), heart rate (F(1) = 61.1; *p* < 0.001; ε^2^ = 0.28 moderate), power (F(1) = 1041.5; *p* < 0.001; ε^2^ = 0.34 moderate), cadence (F(1) = 17.9; *p* < 0.001; ε^2^ = 0.54 moderate), and speed (F(1) = 854.9; *p* < 0.001; ε^2^ = 0.39 moderate).

The MANCOVA also allows us to observe the influence of the interaction of T_awc_ and the elevation gradient on the set of variables and then on each of the variables taken (normalized cadence, normalized power, normalized speed, normalized T_co_, normalized heart rate) individually for each athlete. It is apparent that the interaction between T_awc_ and elevation gradient significantly (*p* < 0.001) influences all variables.

The interaction of T_awc_ and elevation gradient influences T_co_ but less significantly (F(1) = 4.7; *p* < 0.05; ε^2^ = 0.01 very small). Although the interaction does not appear to influence heart rate, the other variables are all significantly influenced: power (F(1) = 87.4; *p* < 0.001; ε^2^ = 0.03 small), speed (F(1) = 57.8; *p* < 0.001; ε^2^ = 0.01 very small) and cadence (F(1) = 22.7; *p* < 0.001; ε^2^ = 0.03 small). 

According to the correlation matrix over the entire race, cadence and speed are correlated to elevation gradient (r = 0.78, *p* > 0.01 and r = −0.95, *p* < 0.01, respectively. However, during uphill sections, cadence does not correlate with elevation gradient (r = −0.17, *p* < 0.05) while speed seems less related to gradient of elevation (r = −0.78 *p* < 0.05). During flat sections, cadence and speed are moderately linked to gradient elevation variation (r = 0.63, *p* < 0.05, and r = −0.59, *p* < 0.05, respectively). Uphill section speed is strongly related to gradient of elevation (r = −0.90 *p* < 0.01) while cadence and gradient are moderately correlated (r = 0.48 *p* > 0.05).

### 3.6. Power Output

**Figure 5:**
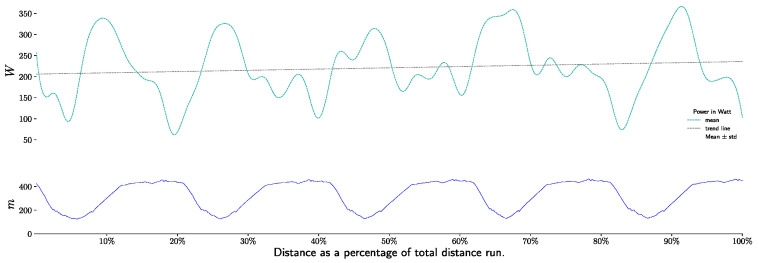
Power output (W), during race, per sections and laps.

**Figure 6:**
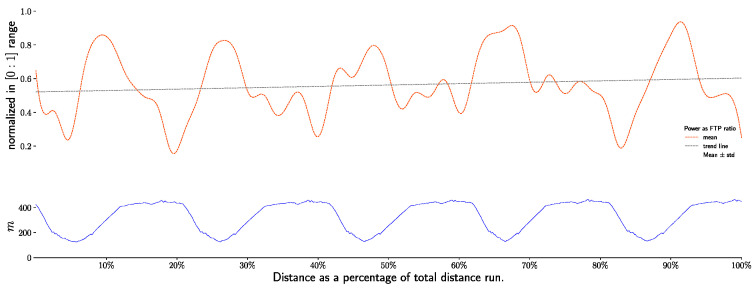
Power output as ratio of FTP test value (W·kg^−1^), during race, per sections and laps.

Power output is significantly different between sections (downhill vs. uphill vs. flat, all pairwise *p* > 0.001) and between laps (1 to 5, *p* < 0.001). During the race, power out drop from 243 +/− 34 watts to 229 +/− 46 watts for uphill, and also during downhill section from 198 +/− 78 to 153 +/− 60. During flat sections, power output rise and drop (Figure 5 and Figure 6).

Table 4 shows the correlation matrix between power output expressed as a ratio of FTP and speed, cadence, heart rate, core temperature, and elevation gradient. The correlation between power and elevation gradient is moderate in all sections, whereas no correlation appears with core temperature. Power is strongly related to heart rate in all sections. In downhill sections, cadence is not related to power, with the correlation dropping to 0.13, whereas in flat and uphill sections, cadence and power are strongly correlated.

## 4. Discussion

The objective of this pilot study was to investigate the impact of a cold environment on the thermoregulatory system, physiological factors and on the performance of professional road cyclists under real ambient temperature exercise conditions (15.60 °C with WCI of 7.77 °C).

### 4.1. Normalized Ambient Temperature (T_awc_) 

With an average ambient temperature of 15.60 ± 1.37 °C corresponding to an average T_awc_ of 7.77 ± 2.04 °C, the race took place in a cold environment. Although the athletes practiced their warm-ups wearing cuffs and a windbreaker jacket, they all abandoned these accessories once the race began and ran wearing a short-sleeved undershirt, a short-sleeved jersey shirt, and jersey shorts as well.

Analysis of the correlation between T_awc_ and speed during the race shows that speed tends to increase as T_awc_ increases. This result is in agreement with the work of Knechtle et al. [44] who show that during a marathon elite male athletes achieve higher running speeds when the temperature is between 8 °C and 15 °C than when it is between 0 °C and 7 °C. 

The impact of T_awc_ (7.77 °C) and air velocity on T_co_ highlighted by our results confirms the results of previous work on cycling by Peiffer and Abbiss [45] and Boynton et al. [46] even though these took place in the laboratory. The study by Oksa et al. [47] also suggests that T_awc_ primarily influences skin and muscle temperature, which in turn impact T_co_. However, T_awc_ does not appear to be a limiting factor in achieving high maximum rectal temperature values. 

Indeed, the maximum core temperature value recorded in the athletes was 39.2 °C. This is on the one hand in agreement with the observations of Ross et al. [48] who observed a maximum core temperature of 38.9 °C under similar non-standardized conditions (13–16 °C). On the other hand, in a warm environment (37 °C), Racinais et al. [6] observe a maximum T_co_ value also of 39.2 °C during a 257.5 km road race. While these same authors note a maximum T_co_ of 41.5 °C during the team time trial (40 km). It is therefore suggested here that the rise in T_co_ depends more on T_awc_ and intensity when the latter fluctuates during endurance exercise, whereas when exercise is of maximum intensity and short duration (less than 15 min), the rise in T_co_ depends more on exercise intensity than on ambient temperature. when exercise is of maximum intensity and short duration (less than 15 min). Thus, we can say that in our study, T_awc_ mainly impacts skin and muscle temperature through air velocity phenomenon which, in turn, impacts T_co_. 

### 4.2. Core Temperature (T_co_)

In our study, athletes were subjected to significant cooling. The mean exercise core temperature of the athletes was lower than the resting core temperature (37 °C) throughout the race (36.58 ± 1.46 °C) (Figure 3). According to some authors [49] a core temperature below 37.5 °C has a negative impact on VO_2max_. However, there is a core temperature [14,23] and an optimal muscle temperature [50,51] for performance that is specific to each athlete and would be around 39 °C for both. 

Work by Ito et al. [52] shows that in athletes, running in an environment with an ambient temperature of 5 °C, simply covering the arms significantly increases skin and core temperature, leading to improved perception of thermal comfort and thermal sensation. The skin temperature (Tsk) varies strongly with the ambient temperature, with an average value of about 33 °C at thermal neutrality, which can drop sharply by exposing the whole body or a certain part of the body to the cold; the tactile sensitivity to cold appears between 15 and 20 °C, the critical temperature for manual dexterity being between 12 and 16 °C. We tried to collect skin temperatures from different parts of the body in our study, but the sensors used did not work well. Nevertheless, given the limited data collected and the work of Ito et al., it would be interesting to recommend to athletes as well as staff to integrate a clothing strategy (partial coverage of certain body areas) depending on the overall event (environmental conditions), and race (race intensity, race terrain, team strategy).

Spitz et al. [53] showed that starting a rowing or running event in a cold environment (5 °C) with skin and core temperatures close to exercise temperature has a positive impact on performance. Indeed, when thermal conditions are cold to neutral, heat input and output are balanced, there is no heat storage and body temperature is balanced. This study highlights the fact that in a cold environment, the longer the time between the end of the warm-up and the start of the run, the more the skin and core temperature drops and the more the performance is negatively impacted. This is reflected in our results since in our study, our athletes only performed a short warm-up on a home trainer before the stage. It would therefore be relevant to implement a warm-up before each stage of a circuit of sufficient duration and intensity in order to increase body and muscle temperature. Thus, the increase in body and/or skin temperature could be used as a marker of the effectiveness of the warm-up. Following the warm-up, it will be important to ensure that the temperature is maintained at an elevated level until the start of the race, through the measurement of the T_co_ associated with a clothing strategy to keep the athlete warm. More insulating clothing strategies could therefore also be proposed. Indeed, a skin temperature and core temperature close to the optimal exercise temperature have a positive impact on performance. 

### 4.3. Heart Rate (HR)

The influence of a cold environment on exercise heart rate has been well documented for many years [21,46,54,55,56]. The effect of skin cooling on heart rate and blood pressure varies depending on the area of the body that is cooled [57,58,59]. In our study, during the descent phases, the normalized ambient temperature (T_awc_) decreases. The face (which contains many thermoreceptors), arms are cooled by convection and the speed of movement, in these phases, the heart rate decreases due to a parasympathetic reflex [57,58,59]. The overall response to cold is visible by the HR and the decrease in T_co_ as the run progresses. However, it is difficult to specify how the cold environment impacts the performance of our athletes because responses vary widely from person to person. Here we see that the variations in normalized ambient temperature (T_awc_) and heart rate (HR) are quite similar. Although at rest, acute exposure to cold causes a hormonal response that increases heart rate, during exercise vasoconstriction in the muscles decreases their perfusion capacity and limits cardiovascular work, leading to a decrease in exercise heart rate. This confirms the significant influence of T_awc_ on HR found in our results. The lower HR in the cold also relates to the smaller body temperature increases, greater dissipation of heat to the environment and lower physiological strain. 

When climbing, the muscular work and consequently the cardiovascular work is more important. With exercise the insulation provided by muscle decreases as blood flow increases. Relative to warmer conditions, muscle blood flow at a given workload may be reduced if deep muscle temperature is below normal (i.e. 39 °C optimum). Respiratory heat loss is often assumed to represent 8% of the total metabolic heat production. However, during exercise this value will increase as minute ventilation increases. Loss of significant amounts of heat from the distal extremities can limit performance, even though the area is not directly involved with exercise [60]. In addition, the normalized ambient temperature (T_awc_) increases as the athlete’s speed decreases. Thus, in addition to the impact of T_awc_ on HR, the altimeter profile of the run is largely involved in the interaction between normalized ambient temperature and heart rate.

### 4.4. The Body Mass

During physical exercise, mental stress, and/or exposure to extreme weather conditions, marked disturbances in body water balance can occur. This is as true in cold climates as it is in hot climates [61,62,63,64]. The results of our study show a measured body mass loss of 1.2% (±0.8%). We can say that this loss of body mass in all our athletes is of sweat and respiratory origin (because no urine or stool emission during the race). This characterizes the existence of dehydration, which is not compensated for by water intake during exercise. Although the importance of hydration on performance in hot climates has been recognized for years, much less is known about the effects of hydration in cold climates. Few studies have specifically evaluated the effects of cold-induced dehydration during exercise, thermoregulation, or performance in cold environments. In fact, neither of the two major review articles that discuss cold water balance specifically address the aspects, implications, or concerns of athletes. Logan-Sprenger [64] observed a 3.3% decrease in body mass during a triathlon race in a temperate environment (20 °C), they conclude that ambient temperature influences body mass losses through sweat and respiratory losses. Although our results are lower than those of Logan-Sprenger [64], the body mass loss measured (1.2% ± 0.8%) in all our athletes is of sudoral and respiratory water loss (no urinary or saddle loss during the race). This mass loss is characteristic of mild dehydration. We know that dehydration leads to an increase in blood viscosity, which in turn increases cardiovascular work. However, in our study, all our athletes had a moderate (or slightly increased) HR during the event, but which corresponds to the values reported in the literature for professional events [1,65]. 

The hypothesis suggested here is that cold decreases HR during endurance exercise, thus counteracting the effect of dehydration on HR. This raises the question of the influence of dehydration on performance. It has often been argued that a water loss >2% of body weight has a negative impact on endurance capacity [66] yet the work of Goulet [67] shows that during outdoor exercise at fluctuating intensity, a loss of 4% of body weight does not alter performance. Furthermore, according to Ebert et al. [62] although a reduction in body weight decreases the power required at the same speed during an ascent (8% gradient), in their experiment this does not translate into better ascent performance. This is what we also find in our study. Of course, in order to strengthen our results, we would have to calculate the water loss to know the real hydration state of our athletes.

### 4.5. The Elevation Gradient 

According to our results, the elevation gradient also influences core temperature. Indeed, our results show that the elevation gradient significantly influences T_co_ and HR. This is consistent with the results of the correlation matrix, which reveals that when core temperature increases, heart rate also increases. These results are in agreement with the study by Bouillod et al. [68], which shows that power is greater over a similar effort (the same power output in incremental testing) when the road gradient is 8% than when it is −0.2%.

Indeed, Bouillod et al. [68] have shown that higher percentages of gradient imply greater muscle work. It is now well accepted that the power of exercise is ~25% (contraction) and ~75% is converted to heat [69,70]. Since the muscle is located between the internal and peripheral compartments, this additional heat production impacts core temperature [71]. According to Tikuisis et al. the muscle acts as a 3rd compartment located between the core and the periphery [71]. Heat induced by skeletal muscle contraction (during exercise or thermal shivering) increases body temperature. This heat production can be used to maintain body temperature when air convection lowers skin temperature. The muscle compartment produces heat that is transferred by blood flow to the central compartment and then to the periphery of the body for removal. The thermodynamic efficiency of muscle contraction results in massive heat production. It can be assumed that at a high heart rate, the conduction time of the increase in muscle temperature must be short since, cold-induced vasoconstriction greatly reduces the flow of warm arterial blood to the region and skin temperature drops. During exercise, neutrally mediated vasoconstriction is inhibited at very low local temperatures, resulting in vasodilation. The resulting influx of warm arterial blood establishes a negative feedback loop (often referred to as ‘hunting reflex’) in which alternating vasoconstriction and vasodilation result in decreases and increases in skin and core temperature. During exercise, the heat flux of the hands and feet increases [72,73], indicating an increase in blood flow. Finger and toe temperatures generally increase and the magnitude of the “flush reflex” decreases with exercise. Skin blood flow thus limits heat transfer from the skeletal muscle to the central compartment during cold exercise [74].

The preservation of core temperature during exercise in cold environments therefore depends on the body’s ability to generate sufficient heat to compensate for heat loss, in the absence of insulating clothing, but also on the ability to transfer the limited heat loss to the central compartment. Secondly, in cycling the regional heat flow is not necessarily constant with the duration, intensity of the exercise, speed of movement and varies considerably [60]. In our study, during the uphill phases, the speed of our cyclists decreased but the power developed increased (and vice versa during the downhill phases), which implies that an additional (muscular) effort was required here. Finally, it should not be forgotten that when exercising in a cold environment with convection, respiratory heat loss and skin heat loss are increased (respectively in the neutral state) thus reducing heat transfer from the muscle compartment to the central compartment via the blood flow. 

In our study, these losses could not be measured. Indeed, we could not record the skin temperature during the run because some of the sensors we attached to the skin (arm, neck, leg) could not be used due to the massive sweat loss. Breathing losses are very difficult to quantify in real race conditions.

### 4.6. Power Output

Bouillod et al. [68] also showed that cadence is significantly higher on a −0.2% gradient compared to an 8% gradient. In our study, the results of the interaction between T_awc_ and elevation gradient has a greater influence on performance factors such as power output, speed, and cadence than on physiological factors (HR) and the thermoregulatory system (T_co_). It would be relevant to conduct further studies in real running conditions integrating skin temperature and muscle temperature to confirm or expand on these results. Because of their duration and the successive repetition of events (stage races), professional cycling competitions generate fatigue, which becomes chronic as the stage and the Tour progress. It has been shown that fatigue can disrupt thermoregulatory mechanisms and thus limit performance. This is known as “thermoregulatory fatigue” [75]. The work of Young et al. [76] shows that chronic fatigue, generated by an intense training program (9 weeks with an average energy expenditure of 4100 kcal), a lack of sleep (average nights of 4 h) and an alteration of the negative energy balance (average intake of 3300 kcal per day), alters the thermoregulatory system in cold. In our study, cyclists were only subjected to a high exercise load. However, the real-life conditions of professional racing mean that strategic aspects must be considered. Indeed, the intensity of the race (power output, cadence and speed) depends on the different tactics used by the professional team. Furthermore, within the teams themselves, the instructions, and therefore the roles, of each cyclist is different, thus influencing the study results obtained. This implies that all athletes have an intensity management that depends on both measurable factors (e.g., physical capacity, environment) and non-measurable factors (e.g., role, team strategy, individual strategy) that are difficult to take into account in the analysis of our results.

## 5. Conclusions

The context of the study, i.e. the measurement of thermal and physiological parameters in real cycling race conditions within a professional team, was an opportunity but did not allow us to acquire a wider range of data (such as the measurement of skin temperature, muscle temperature or the collection of comfort and thermal sensation questionnaire during the race). Indeed, the race regulations impose certain constraints such as a maximum number of seven athletes per team (in our case, one of the seven athletes in our study withdrew after a serious fall in the time trial the day before the study), or a limitation on the type of equipment carried on the athlete, which limits the number of athletes who can participate in the study as well as the type of study carried out, and therefore the analysis of our results is also affected. 

However, the results of our primary study allow us to affirm that in cold environmental conditions, the athletes studied were not able to perform at their maximum potential mainly during the phases of climbing the circuit performed five times. 

In fact, the interaction between normalized ambient temperature and the elevation gradient significantly modifies thermal (core temperature), physiological (heart rate) and performance factors (power output, speed and cadence). 

If we were to make strategic recommendations for professional cyclists who must perform in cold ambient conditions, we would advise the following measures: (1) implementing training and practice strategies to provide performance experience in cold environments and to acclimatize to the cold by training in the same conditions to those that may be encountered in competition, (2) rethinking pre-race warm-up methods to allow athletes to increase their internal and muscle temperature prior to the start by reaching an optimal temperature, (3) adapting exercise drinks to the ambient temperature to optimize the hydration of the athletes in these conditions, (4) advocating a better management of clothing (more insulating clothing) to avoid the cooling of the skin and the muscles before the start of the race and during the race.

In perspective, we plan to reproduce our study by including the measurement of skin temperature during several stages of a grand tour in different environmental conditions (hot, cold, high elevation gradient, sea level). 

## Figures and Tables

**Table 1:** Stage characteristics by section.

Section	Distance	Elevation Gain	Mean Elevation	Maximal Elevation
Stage	181.00 km	2315 m		
Track	29.80 km	353 m		
main climb	2.80 km	226 m	8%	15.1%

**Table 2:** Time, heart rate, speed, power, cadence, elevation gradient, ambient temperature, normalized temperature and body core temperature during the race by laps and per sections Data are presented as mean value and standard deviation.

Lap	Section	Time (min)	Temperature (°C)	Wii (°C)	Gradient of Elevation (m·s^−1^)	Core Temperature (°C)	Heart Rate (Bpm)	Power (W)	Speed (mps)	Cadence
1	Downhill	12.55 ± 0.36	16.71 ± 0.76	8.16 ± 2.7	−0.4 ± 0.42	37.25 ± 0.59	127.76 ± 16.02	143.63 ± 54.34	13.44 ± 4.41	53.0 ± 13.34
	Uphill	14.34 ± 0.5	16.93 ± 0.66	11.41 ± 2.39	0.06 ± 0.19	36.66 ± 0.72	146.45 ± 13.61	296.47 ± 55.86	6.42 ± 1.94	81.14 ± 7.53
	Flat	13.89 ± 0.36	16.24 ± 0.82	7.64 ± 1.53	0.32 ± 0.23	36.97 ± 0.65	136.64 ± 10.38	206.71 ± 35.81	11.16 ± 0.88	80.0 ± 6.07
	*Lap*	40.78 ± 0.41	*16.63 ± 0.8*	*9.11 ± 2.81*	0.01 ± 0.32	*36.95 ± 0.7*	*137.28 ± 15.43*	*218.23 ± 79.54*	*10.23 ± 4.01*	*72.03 ± 15.82*
2	Downhill	12.47 ± 0.31	149.90 ± 8.32	7.72 ± 1.34	−0.41 ± 0.38	36.43 ± 1.0	112.97 ± 10.26	111.9 ± 50.71	13.91 ± 2.37	46.92 ± 16.15
	Uphill	14.24 ± 0.31	16.43 ± 0.55	10.94 ± 2.28	0.02 ± 0.21	36.50 ± 1.87	148.37 ± 9.47	295.51 ± 41.93	6.29 ± 2.32	83.09 ± 5.64
	Flat	20.78 ± 0.08	16.42 ± 0.86	8.01 ± 1.5	0.33 ± 0.22	35.82 ± 1.98	130.79 ± 8.95	184.43 ± 32.84	10.98 ± 1.06	73.99 ± 7.09
	*Lap*	47.49 ± 0.21	*16.52 ± 0.72*	*8.80 ± 2.22*	0.01 ± 0.32	*36.18 ± 1.76*	*131.26 ± 16.29*	*198.05 ± 80.89*	*10.37 ± 3.47*	*69.49 ± 17.32*
3	Downhill	11.88 ± 0.28	16.40 ± 0.49	6.81 ± 1.13	−0.43 ± 0.42	36.74 ± 1.06	130.07 ± 17.05	173.08 ± 77.91	14.88 ± 2.03	60.88 ± 15.89
	Uphill	13.79 ± 1.72	16.64 ± 0.8	11.32 ± 2.47	0.02 ± 0.23	36.47 ± 1.37	149.90 ± 8.32	277.37 ± 42.56	6.14 ± 2.29	80.55 ± 5.56
	Flat	20.38 ± 0.08	16.41 ± 0.87	7.83 ± 1.58	0.32 ± 0.21	36.54 ± 1.34	138.08 ± 10.53	201.07 ± 39.62	11.27 ± 1.0	75.5 ± 9.29
	*Lap*	46.05 ± 0.69	*16.48 ± 0.78*	*8.64 ± 2.58*	0.01 ± 0.32	*36.57 ± 1.29*	*139.66 ± 14.18*	*217.3 ± 67.23*	*10.62 ± 3.75*	*73.31 ± 13.03*
4	Downhill	11.23 ± 0.27	15.99 ± 0.48	5.97 ± 1.13	−0.45 ± 0.43	36.67 ± 1.02	149.65 ± 15.07	223.52 ± 75.18	15.78 ± 2.04	66.0 ± 10.5
	Uphill	12.92 ± 1.1	15.95 ± 0.46	9.80 ± 2.5	0.01 ± 0.23	37.17 ± 1.34	170.33 ± 7.75	345.49 ± 24.59	7.14 ± 3.05	83.62 ± 6.27
	Flat	26.29 ± 1.18	15.38 ± 0.97	6.24 ± 1.64	0.37 ± 0.24	36.09 ± 1.94	145.74 ± 12.2	213.58 ± 31.8	11.48 ± 0.98	76.65 ± 8.15
	*Lap*	50.44 ± 0.85	*15.66 ± 0.83*	*7.05 ± 2.39*	0.01 ± 0.32	*36.49 ± 1.69*	*152.66 ± 15.77*	*248.19 ± 71.18*	*11.4 ± 3.55*	*75.91 ± 10.36*
5	Downhill	12.63 ± 0.58	14.56 ± 0.5	4.21 ± 1.09	−0.4 ± 0.39	36.07 ± 1.60	124.3 ± 15.39	126.1 ± 55.98	14.04 ± 2.14	54.27 ± 14.11
	Uphill	14.48 ± 1.61	14.73 ± 0.44	8.34 ± 2.14	0.04 ± 0.22	36.7 ± 1.11	155.25 ± 14.45	315.11 ± 46.25	6.76 ± 2.32	81.49 ± 6.14
	Flat	16.06 ± 1.08	13.28 ± 0.81	3.46 ± 2.06	0.31 ± 0.21	36.40 ± 1.13	142.41 ± 18.99	199.14 ± 51.84	10.92 ± 1.51	76.24 ± 11.89
	*Lap*	43.17 ± 1.09	*14.11 ± 0.92*	*5.18 ± 2.82*	0.01 ± 0.32	*36.79 ± 1.40*	*140.93 ± 20.58*	*212.83 ± 90.46*	*10.58 ± 3.47*	*71.27 ± 15.97*
Overall Race		227.93 ± 1.37	15.60 ± 1.37	7.77 ± 2.04	0.01 ± 0.32	36.58 ± 1.46	140.54 ± 18.03	219.33 ± 79.72	10.66 ± 3.67	72.47 ± 14.79

**Table 3:** Correlations between T_awc_ and power, power as ratio of ftp, speed, cadence, heart rate and core temperature., expressed by r value (rounded to two digits, significativity ** *p* < 0.01, *** *p* < 0.001).

T_AWC_	Power	Power as FTP	Speed	Cadence	Heart Rate	Core Temperature
Downhill	0.04 ***	0.04 ***	−0.56 **	−0.02 **	−0.05 ***	0.31 **
Uphill	0.30 ***	0.28 **	−0.84 **	−0.43 **	0.18 **	−0.11 **
Flat	0.04 **	−0.03 ***	0.43 **	−0.19 **	−0.11 **	−0.07 ***

**Table 4:** Correlation between Power as ratio of FTP and speed, cadence, heart rate, core temperature and gradient of elevation, per sections. ** *p* < 0.01, *** *p* < 0.001.

Power as Ratio of FTP Test	Speed	Cadence	Heart Rate	Core Temperature	Gradient of Elevation
Downhill	−0.42 **	0.13 ***	0.66 **	0.020 **	0.67 **
Uphill	−0.10 **	0.89 **	0.87 **	0.080 ***	0.43 ***
Flat	0.029 **	0.65 **	0.69 ***	0.12	0.43 **

## Data Availability

All data and results are stored by the study investigators.
